# Selective effects of serotonin on choices to gather more information

**DOI:** 10.1177/0269881121991571

**Published:** 2021-02-18

**Authors:** James JA Livermore, Clare L Holmes, Jo Cutler, Maruša Levstek, Gyorgy Moga, James RC Brittain, Daniel Campbell-Meiklejohn

**Affiliations:** 1 Sussex Neuroscience/School of Psychology, University of Sussex, Brighton, UK; 2 Donders Institute for Brain, Cognition and Behaviour, Radboud University, Nijmegen, The Netherlands; 3 School of Psychology, University of Birmingham, Birmingham, UK; 4 Brighton and Sussex Medical School, Brighton, UK; 5 Chelsea and Westminster Hospital, London, UK

**Keywords:** Information sampling, decision making, serotonin, citalopram, atomoxetine, risk preference

## Abstract

**Background:**

Gathering and evaluating information leads to better decisions, but often at cost. The balance between information seeking and exploitation features in neurodevelopmental, mood, psychotic and substance-related disorders. Serotonin’s role has been highlighted by experimental reduction of its precursor, tryptophan.

**Aims:**

We tested the boundaries and applicability of this role by asking whether changes to information sampling would be observed following acute doses of serotonergic and catecholaminergic clinical treatments. We used a variant of the Information Sampling Task (IST) to measure how much information a person requires before they make a decision. This task allows participants to sample information until satisfied to make a choice.

**Methods:**

In separate double-blind placebo-controlled experiments, we tested 27 healthy participants on/off 20 mg of the serotonin reuptake inhibitor (SRI) citalopram, and 22 participants on/off 40 mg of the noradrenergic reuptake inhibitor atomoxetine. The IST variant minimised effects of temporal impulsivity and loss aversion. Analyses used a variety of participant prior expectations of sampling spaces in the IST, including a new prior that accounts for learning of likely states across trials. We analysed behaviour by a new method that also accounts for baseline individual differences of risk preference.

**Results:**

Baseline preferences demonstrated risk aversion. Citalopram decreased the expected utility of choices and probability of being correct based on informational content of samples collected, suggesting participants collected less useful information before making a choice. Atomoxetine did not influence information seeking.

**Conclusion:**

Acute changes of serotonin activity by way of a single SRI dose alter information-seeking behaviour.

## Introduction

How does one decide they have enough information to make a decision? Efficient sampling and evaluation of environments enhance the accuracy, efficiency and net benefit of choices, though this can come at the cost of energy, forgone opportunity and, in many species, exposure to harm and predation. Problems from sampling too little or too much information occur in a variety of psychopathologies. Examples include ‘jumping to conclusions’ with insufficient information in psychosis (Dudley et al., 2016), and sampling less information in depression (Tavares et al., 2007), Parkinson’s patients (Djamshidian et al., 2013), substance use disorders (Clark et al., 2006) and binge drinking (Banca et al., 2016). Increased information gathering is observed in patients with obsessive-compulsive disorder and individuals high on the compulsivity spectrum (Hauser et al., 2017a, 2017b). Formal experimental models of these biases provide insight into decision processes associated with these disorders and new targets for their treatments.

Information sampling relates to the psychological construct of *reflection impulsivity* – the tendency to make decisions without gathering and effectively evaluating information. Reflection impulsivity is dissociable from motor and temporal impulsivity forms (Caswell et al., 2015). In recent decades, it has been studied with the Information Sampling Task (IST; Clark et al., 2006) and urn tasks (FitzGerald et al., 2015). In each, participants choose a quantity of information to sample, at a defined cost, before making a decision that will lead to a reward or penalty. The IST is designed to place negligible demands on visual processing and working memory, and have an intuitive interface (Clark et al., 2006). It has multiple trials, each with a new grid of 25 obscured squares concealing one of two colours. For each grid, a participant chooses to reveal as many squares as they wish until certain enough to make a choice of which of the two colours is in the majority for the board. Each sample detracts from the potential reward (in the decreasing win condition of the IST) but increases the likelihood of a correct decision. These two competing accounts must strike an optimal balance. The switch from sampling to colour prediction relates to the explore–exploit learning theory framework. An individual explores the information available before committing to a choice by exploiting this information (Averbeck, 2015).

Serotonin has received attention for links to various concepts related to information sampling: impulsivity in general (Dalley and Roiser, 2012), avoidance of aversive outcomes (Cools et al., 2011) and risk preferences (Murphy et al., 2009). Murphy et al. showed that tryptophan supplementation reduced loss aversion in probabilistic decision making, while acute tryptophan depletion (ATD) manipulations have been shown to have effects on risk preferences in rats (Koot et al., 2012) and macaques (Long et al., 2009). Crockett et al. (2012) specifically examined the effects of ATD on IST behaviour. Participants increased costly sampling when tryptophan (serotonin’s precursor) was depleted, while sampling without cost was unaffected. It was posited that serotonin may reduce avoidance of local costs relative to the prospect of a future global loss. Similarly, in other contexts, ATD has been shown to increase deferral of complex decisions to make purchases (Lichters et al., 2016). Comparable studies using clinical doses of serotonergic medication have not been reported. Serotonin reuptake inhibitors (SRIs) blockade the serotonin transporter and inhibit serotonin’s reabsorption into the presynaptic neuron, and are widely used drugs for psychopharmacological treatment of depression and other psychopathologies. Citalopram is a highly selective SRI (Owens et al., 2001). Chamberlain et al. (2006) reported effects of acute citalopram on probabilistic reversal learning. That research showed that a single 30 mg dose of citalopram impaired learning about a changing reward environment, increasing sensitivity to negative feedback. This line of evidence led to our hypothesis that serotonin, by way of a clinical dose of citalopram, may also change learning in information sampling contexts.

To contrast serotonergic effects with effects of other monoamines and employ an active control with a similar side-effect profile, we also tested a group with atomoxetine at a dose known to have behavioural effects. Atomoxetine is a specific noradrenaline reuptake inhibitor that increases prefrontal noradrenaline and dopamine levels (Bymaster et al., 2002; Koda et al., 2010; Swanson et al., 2006) and is associated with improved inhibitory control (Chamberlain et al., 2009; Rae et al., 2016). It has been linked to lowered motor and temporal impulsivity (Bizot et al., 2011; Chamberlain et al., 2006), but not the reflection subtype. The Chamberlain et al. (2006) study showed an effect of citalopram on reversal learning but no effect of 40 mg atomoxetine, despite this dose being shown to enhance inhibitory control (Chamberlain et al., 2009), reduce fatigue and alter subjective sensations (DeVito et al., 2017). This dose also affects affect random (but not directed) exploration in humans (Warren et al., 2017).

Recent advances have also reformulated our modelling of IST decisions. From the point of view of the decider, the probability of a decision being correct on the IST with a given amount of information can be formalised as a Bayesian inference problem (Bennett et al., 2016; FitzGerald et al., 2015). Bayes’ theorem determines the optimal way to combine information from both the current environment (the likelihood) and previous experience (the prior). In the IST, this corresponds to a likelihood based on information available from the number of squares revealed of each colour on the current trial and a prior from true distributions of colours revealed on previous trials. A normative set of choices (an ideal observer) from these models can formulate an upper bound on an individual’s ability to predict outcomes effectively from gathered information. As real-life decision making is often suboptimal, this allows a quantification of how an individual or group departs from the ideal – a measurement of how effectively they can use existing information when deciding whether they have enough. While Bayesian approaches have been used to analyse decisions on this task (Axelsen et al., 2018; Bennett et al., 2016), previous studies have not incorporated updating of prior expectations from previous trials. Participants are often instructed that each sampling board is independent of previous ones. However, it is our assumption that learning of likely underlying colour distributions from previous trials still occurs, especially as repeated trials inevitably reveal that more extreme underlying distributions (where the great majority of squares are of one colour) never occur.

We developed a variant of the IST designed to provide a clearer interpretation of the effects of pharmacological challenges on information sampling decisions. This IST variant uses purely positive or zero pay-offs to remove the possible interpretation of changes of loss aversion with respect to the net result of each trial. Use of the net result as the unit of measurement is a reasonable approach, given the short duration of the trial. However, aversion to local costs of a sample is still a possibility. We also introduced a fixed interval between the sampling and decision periods (rather than the participant choosing to decide at any point in time as in the original task) in order to negate temporal discounting interpretations (i.e. the tendency to sample less through impatience for receiving reward).

Finally, we introduced a new dependent measure that merges a normative view of decision making (i.e. whether it is better or worse from the point of view of the decider) with individual differences of risk preference. Previous studies of the IST (e.g. Chamberlain et al., 2007; Clark et al., 2006; Crockett et al., 2012) used a measure of ‘probability of correctness’ to determine differences between healthy and patient populations or between participants on placebo and pharmacological manipulations. While this is an important aspect of a correct choice, it is limited in its ability to form a normative view. Differences in propensity to accept a given likelihood of being correct (or sample more) cannot be assessed for their effectiveness unless a cost–benefit of sampling or choosing a colour can be formulated, incorporating changes in the net available reward (recall that each sample is costly). A simple approach could assume that decision makers seek to maximise expected value – the net reward available multiplied by the probability of being correct (determined by Bayesian inference). However, considerable evidence shows that most decision makers also exhibit risk aversion (Arrow, 1971; Dohmen et al., 2011; Holt and Laury, 2002) – we tend to prefer more certainty, even if that reduces our average pay-off. Expected utility is the subjective value of a gamble, that is, the value that an individual places on it based on their own preferences, incorporating their personal risk aversion (Von Neumann and Morgenstern, 1944). Changes in expected utility can therefore be seen as changes in the subjective value of a decision in relation to the individual’s preferences (assuming they remain fixed). So, to model biases in models of reflection impulsivity, a normative conceptual framework should measure the expected utilities of participant choices.

For this study, we measured risk preferences in an independent task to incorporate individual differences of risk aversion in our analysis and calculate estimate individual expected utilities. We used the same baseline measure of risk preference in both conditions. Thus, this was not used to establish drug effects on risk aversion, and changes of choice expected utilities could also reflect effects of the treatment on underlying risk preference. In addition to expected utility, we used the probability of being correct for each choice, as in previous studies. In either case, the central dependent measures of this study reflect that participants take into account the informational content of the samples (i.e. how many of one colour compared to the other) to make their decision to sample more or not (Axelsen et al., 2018; Clark et al., 2006). Each dependent measure was determined by two established models and by a new model that incorporates experience with previous trials.

We predicted that citalopram would reduce the efficient use of information presented, in line with effects on learning in other environments. This would be demonstrated by lower expected utility outcomes and probability of being correct for decisions under citalopram compared to placebo. Conversely, unlike citalopram, we predicted that atomoxetine would not affect information sampling. As IST sampling is a directed strategy, that is, a strategy seeking information that can be used to obtain future reward (Wilson et al., 2014), no effect of atomoxetine would be in line with its lack of effect on directed exploration (Warren et al., 2017).

## Methods

### Participants

Ethical permission was granted by University of Sussex Sciences and Technology C-REC (ER/JL332/6, ER/JL332/7). Potential subjects were screened with a health questionnaire (see Supplemental Material) and the Mini International Neuropsychiatric Interview (MINI; Sheehan et al., 1998). Exclusion criteria included: age <18 or >35 years; history of psychiatric disorder (including anxiety disorder, depression, eating disorder, psychosis and substance abuse disorder); presence of significant ongoing medical condition (including migraine, diabetes, epilepsy, glaucoma and hypertension); pregnancy or breastfeeding; currently taking any medication (excluding contraceptive pill); first-degree family history of bipolar disorder; or MINI current indication of major depressive episode, manic episode, panic disorder, social phobia, OCD, PTSD, alcohol dependence, substance dependence, mood disorder with psychotic features, psychotic disorder, anorexia nervosa, bulimia nervosa, generalised anxiety disorder or antisocial personality disorder. Participants were also instructed to abstain from alcohol or caffeine in the 12 hours preceding the start of test sessions.

Fifty-three healthy subjects aged 18–35 years were recruited for this study: 28 for the citalopram group and 25 for the atomoxetine group. Of those, one from the citalopram and two from the atomoxetine group did not complete the study due to adverse side effects, and the data of one participant from the atomoxetine group were excluded, as they took no samples in all but one of the trials. This left 49 participants: 27 for the citalopram group (11 males, *M*_age_ = 23.4 years, *SD* = 4.70 years) and 22 for the atomoxetine group (10 males, *M*_age_ = 23.1 years, *SD* = 2.93 years). The groups (citalopram and atomoxetine) were tested consecutively, and participants were aware in advance which group they were being recruited into. The groups were matched for age and sex. Subjects were tested on two sessions at least seven days apart (days between sessions *M* = 9.60, *SD* = 4.26). Assignment to treatment order was double-blind and counterbalanced, with the drug treatment administered in one session and the placebo in another.

### Procedure

Participants gave informed consent prior to the commencement of the first testing session, and were approved by a medical doctor who assessed blood pressure, heart rate and medical history.

Doses in the drug treatment conditions consisted of 20 mg citalopram/40 mg atomoxetine. These doses have been shown to elicit cognitive changes in previous studies (Browning et al., 2007; Chamberlain et al., 2009; Grillon et al., 2007; Warren et al., 2017), and were chosen to balance active drug effects of interest against the potential for undesirable side effects.

Drug and placebo doses were delivered in gelatine capsules, indistinguishable from one another, with the capsule filled with microcrystalline cellulose (in addition to the active drug in the drug conditions). Drug and placebo doses were all manufactured according to Good Manufacturing Practice guidelines. No one who had contact with participants was aware of the treatment order, which was pseudo-randomised, balanced for sex and coded by a researcher who was not present during testing.

During the first session, following drug administration, participants completed the risk preference elicitation (RPE) task immediately after the first dose but before the treatment was absorbed. They were also given visual analogue scales (VAS) at three time points: immediately following the dose, preceding the start of tasks and following the end of tasks. Scales (from 0 to 100) were given to assess three somatic effects (nausea, headache and dizziness) and five emotion/arousal-related effects (pairs of antonyms: alert–drowsy, stimulated–sedated, restless–peaceful, irritable–good humoured, anxious–calm) to measure whether the drug was affecting these measures. To allow for drug levels to reach peak absorption (Milne and Goa, 1991; Sauer et al., 2005), the citalopram group commenced behavioural testing after three hours from the drug/placebo dose, and the atomoxetine group after one-and-a-half hours (aside from the RPE task – see below). They then carried out a set of tasks, including the Modified IST (mIST). Following the end of behavioural testing and the final scales, participants in the atomoxetine group were monitored for a further one-and-a-half hours, resulting in the same length of testing session for each group.

### Tasks

#### RPE task

This task, adapted from Eckel and Grossman (2002, 2008), was used to elicit the risk preferences of participants. Participants were shown a list of gambles as in Table 1 and told to select their preferred gamble. They were informed that at the end of the session, a fair coin would be flipped to determine whether they received the low or high pay-off. The amount won was added to their participation fee. The choice of gambles was used to calculate a parameter of risk aversion *r* (details in Measures and Analyses). This measure was only given on the first session at the point immediately after drug/placebo administration, and so was used solely as a measure of baseline risk preference (i.e. unaffected by drug manipulation).

**Table 1.** Gamble choices in risk preference elicitation task.

Gamble	Low pay-off	High pay-off	*r* range	*r* value interpolated/extrapolated
1	£1.15	£1.15	*r* > 4.28	5.78
2	£1.00	£1.50	2 < *r* < 4.28	2.97
3	£0.90	£1.80	0.861 < *r* < 2	1.32
4	£0.80	£2.00	0.382 < *r* < 0.861	0.61
5	£0.30	£2.90	−0.317 < *r* < 0.382	0.06
6	£0.05	£3.00	*r* < −0.317	−0.74

#### mIST

**Figure 1.**
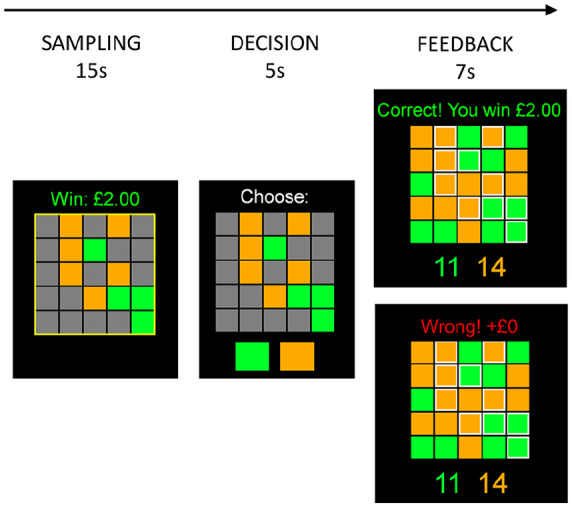
Time course of task.

We designed a modified version of the IST (Clark et al., 2006). Subjects were presented with a grid of 25 grey boxes, which concealed underlying squares in one of two colours, and they were told to open as many as they wished within a 15-second interval before deciding which of the two was in the majority. Each sample taken had a fixed cost, with a positive pay-off for a correct decision and no pay-off for an incorrect one. Ten trials were presented, with no instruction to the subject as to the underlying distribution of the numbers or locations of squares. To make the decision-dependent nature of task winnings more salient, potential winnings in monetary form (starting from £3.00 and decrementing by £0.10 for each sample taken) were displayed to subjects during their sampling decisions. These operated solely in the gain domain (i.e. an incorrect decision resulted in no change, and a correct decision resulted in a positive pay-off) to understand whether serotonergic effects were present where loss aversion was minimised (though loss aversion may still be present if zero outcomes are interpreted as loss). The sampling time was fixed at 15 seconds, with a decision required at that time regardless of how many samples were taken in order to eliminate strategies to reduce task duration/time to reward. The true generative probability was a discrete uniform distribution of the majority colour occupying between 13 and 16 squares. Participants were informed in advance that the winnings from one of the trials selected at random would be paid to them at the end of the study. The time course of the task is shown in Figure 1.

To test that sampling behaviour on the IST variant was similar to previous versions, we tested the mean number of samples against these values in five papers from the literature (Chamberlain et al., 2007; Clark et al., 2006, 2009; Delazer et al., 2011; Tavares et al., 2007) and *p(correct)* where available, using Welch’s *t*-test (Supplemental Table S2).

### Measures and analyses

#### RPE task

The choice of gamble was used to determine individual risk preference parameter *r* of their utility functions in the form of a parameter of constant relative risk aversion (CRRA; e.g. Arrow, 1971; Chiappori and Paiella, 2011). The expected utility of a gamble *G* for any *r*, given the high and low outcomes and an equal probability of 0.5 for each, is:

(1) $$EU_{G}=\left(0.5\frac{{G_{HIGH}}^{1-r}}{1-r}+0.5\frac{{G_{LOW}}^{1-r}}{1-r}\right)$$

The value of *r* at the indifference point between each pair of adjacent gambles was calculated, that is, where the utility of the two gambles was equal (*EU_G_* = *EU_G_*_ + 1_). When individuals chose gambles 2–5, the gamble can be considered as chosen over the gambles on either side. So, the indifference points mark the upper and lower bounds of the individual’s *r*. With gambles 1 and 6, only a lower and upper bound, respectively, are specified. Through non-linear interpolation and extrapolation from those ranges, a point estimate of *r* for each participant was determined. This was used to calculate expected utilities for the behaviour in the mIST.

#### mIST

We computed a set of measures to index behaviour in this task based on and extending Bayesian models of the probability of making a correct choice based on information available – a measure termed *p(correct)* in the literature. This measure captures the fact that the amount of information conveyed by a given number of samples can vary depending on what those samples reveal. For example, a trial with five samples in a 3:2 split of revealed colours conveys much less information about the true majority than if all five are of a single colour. Behaviour in line with maintaining an acceptable level of risk of an incorrect decision should therefore be driven by the amount of information acquired rather than a set number of samples – taking fewer samples when the overwhelming majority is one colour compared to when they are more mixed. For this reason, *p(correct)* has been considered a more useful quantification of task behaviour than sample number in the existing literature (Clark et al., 2006, 2009; Delazer et al., 2011; Tavares et al., 2007).

To test formally that participants were guided by the informational content of samples rather than their quantity, we performed an additional analysis to measure the consistency of behaviour across trials according to the measures. Consistent behaviour on a measure is taken as an indication that participants are more likely to be guided by information represented by that measure (e.g. *p(correct)* vs. number of samples). We used coefficients of variation (CVs), defined as the measure’s ratio of standard deviation to its mean within each participant’s data. CVs are used to assess the consistency of measure on ratio scales with different mean values (Quan and Shih, 1996; Shechtman, 2013). A lower CV indicates greater consistency. We computed a CV for each participant on each measure (*p(correct)*, expected utility and sample number), averaging the former measures across the three prior models described in the following paragraph (see Supplemental Table S1). We compared CVs of *p(correct)* and expected utility to CVs of sample number using paired *t*-tests. These tests confirmed the appropriate use of *p(correct)* and expected utility.

We then considered that since there were relatively few trials and the distribution was not specified to subjects in advance, there is little experience to build up an accurate prior, and the discrete structure has few possible outcomes. So, it is feasible for participants to keep approximate track of the outcomes, deliberately or otherwise. We sought a principled approach to accounting for this possibility in our analysis that did not require a complex inference and made minimal assumptions. This was the categorical distribution, where observed frequencies of outcomes added to the probability mass for the prior of the next decision. We refer to this as the learned prior model of choice probability and value. To solve the problem of the unknown personal prior (i.e. the participant’s personal a priori interpretation of the probability structure of the task) for the first trial in which no experience had been gained of the board, data from the first trial were excluded from analysis. So, no information or distributional assumptions beyond the feedback presented and the current trial’s information set was required.

Evidence has shown that the choice of prior can affect statistical inferences on data (Bennett et al., 2016). We wished to remain agnostic about which priors were held by individuals. So, we also computed probabilities for two other established models (excluding the first trial for all to allow for comparison, as the measure was undefined for the learned prior model). In all, we examined behaviour using the learned prior model (described above), the binomial prior model (Axelsen et al., 2018; Clark et al., 2006) and the flat prior model (Bennett et al., 2016). Full details of the three models are given in the Supplemental Methods.

Potential rewards were transformed into expected utilities (*EU*) using a utility function with CRRA. As the equation required strictly positive inputs, a constant *c* was also added prior to transformation, which was the cost of a sample (the smallest unit of outcome). The resulting utilities were then normalised so that the utilities of the maximum and minimum possible rewards (£3 and £0) were set to 1 and 0.

$$U^{\prime }\left(reward\right)=\{\begin{array}{c} \frac{{\left(reward+c\right)}^{1-r}}{1-r}if\;r\neq 1\\ \log\left(reward+c\right)if\;r=1 \end{array}$$

$$U\left(reward\right)=\frac{U^{\prime }-\min\left(U^{\prime }\right)}{\max\left(U^{\prime }\right)-\min\left(U^{\prime }\right)}$$

EU(reward)=p(correct).U(reward)

To allow for comparison with past studies, we performed statistical tests on both *EU* and *p(correct)* measures, calculating each measure for the three prior models. Tests within each group were performed in SPSS v25.0 (IBM Corp., Armonk, NY) using paired-samples *t*-tests, and between groups with repeated-measures analyses of variance (ANOVAs; all two-tailed). A significance level of α = 0.05 was used throughout.

To test whether somatic effects of the drugs were potentially a source of changing performance, we used the VAS measures and mediation analyses. Details are given in the Supplemental Material.

## Results

### Self-report VAS measures

There was a small but significant difference in the nausea scale between drug and placebo conditions for atomoxetine and a difference on the threshold of significance for citalopram, corresponding to a change of 3.9 (citalopram)/6.6 (atomoxetine) on a 100-point scale. We conducted a supplementary mediation analysis to determine whether the change in nausea might account for effects of drugs on the dependent measures of the mIST. Changes of nausea did not predict any mIST-dependent measure, and all mediation effects via nausea were non-significant. There were no significant differences between the two drugs in drug–placebo differences. See Supplemental Material for full details of these analyses.

### RPE task

The results of the RPE (Table 2) show that 20 out of 27 participants in the citalopram group and 15 out of 22 participants in the atomoxetine group showed a degree of risk aversion (choosing gambles 1–4), while three and two, respectively, were approximately risk neutral (gamble 5) and four and five, respectively, were risk seeking (gamble 6). Weighted mean gambles were 3.11 for the citalopram group and 3.77 for the atomoxetine, indicating a baseline (pre-dose effect) preference for slightly higher risk in the atomoxetine group. However, Fisher’s exact test showed that the difference in proportions of each gamble choice between the groups was not significant (*p* = 0.37).

**Table 2.** Numbers and proportions of participants choosing each gamble in the RPE task.

Gamble	Citalopram	Number of choices		Proportion of sample		
		Atomoxetine	Total	Citalopram	Atomoxetine	Total
1	5	0	5	0.19	0	0.10
2	9	7	16	0.33	0.32	0.33
3	2	3	5	0.07	0.14	0.10
4	4	5	9	0.15	0.23	0.18
5	3	2	5	0.11	0.09	0.10
6	4	5	9	0.15	0.23	0.18

RPE: risk preference elicitation.

### mIST

The CV was lower for both *p(correct)* and expected utility than for the number of samples in both drug and placebo conditions (see Supplemental Material for details). This indicated that participants used the information within samples rather than the number of samples to guide behaviour.

Average *p(correct)* and number of samples taken on the mIST was similar to prior studies. Specific values of *p(correct)* in previous studies was only available in one paper by Clark et al. (2009), with other papers using the measure in statistical analyses and displaying results in chart form. Clark et al. found a similar *p(correct)* value (using a binomial prior) in the healthy control group to the equivalent mean placebo value (also binomial prior) across both drug groups in our study (this study: *M* = 0.720, *SD* = 0.054; Clark et al., 2009: *M* = 0.74, *SD* = 0.06; *t*(30.03) = −1.27, *p* = 0.21). The mean sample number was 8.37 (*SD* = 3.89), which was not significantly different to the weighted mean of previous studies (*M*_weighted_ = 8.84, pooled *SD* = 3.83; *t*(81.1) = 0.74, *p* = 0.46; details of included studies are given in Supplemental Table S2).

We transformed each participant’s decision utilities using equations 1–3, with the individual implied utility function parameters computed from the RPE task used as the participant’s *r* parameter. These were computed separately using the three probability models: binomial prior, flat prior and learned prior. Treatment order and sex were tested for effects on all dependent measures. Neither showed significance or trend significance when tested as a between-subjects factor for any group comparisons, and so they were excluded from the final analysis.

**Figure 2.**
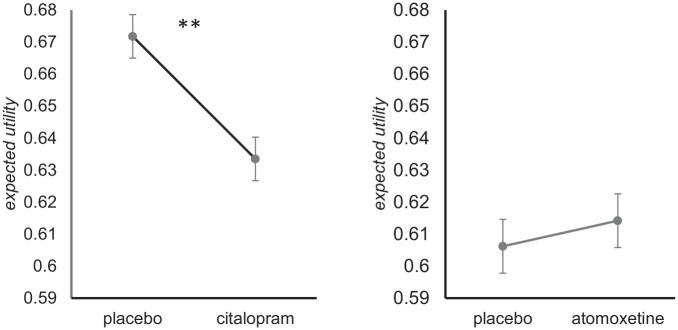
Expected utilities and probability of correct response under learned prior model. Error bars are ±1 standard error of the mean. **p <0.01.

The three expected utility derivations were highly correlated (corr(*EU*_learned_, *EU*_flat_) = 0.950, corr(*EU*_learned_, *EU*_binomial_) = 0.980, corr(*EU*_flat_, *EU*_binomial_) = 0.970; all *p* < 0.001). Comparing within-subjects means of each score by drug treatment, significant differences were shown between placebo and citalopram for all three prior models of expected utility (Table 3). This provided strong rejection of the null hypothesis of no drug effect. In each case, the mean expected utility of decisions made under citalopram was lower than that of those made under placebo. By contrast, those between placebo and atomoxetine were non-significant (Table 4). These results are shown in Figure 2. An ANOVA across both sets of data, with drug group as a between-subjects factor and drug session as a within-subjects factor, also showed a significant drug group × session interaction on learned and flat prior models, and trend significance on the binomial prior model (Table 5).

**Table 3.** Results of within-subject comparisons of drug and placebo conditions for the citalopram group.

	Statistic	Expected utility		*p(correct)*	
		Placebo	Drug	Placebo	Drug
Learned prior	*M* (*SD*)	0.672 (0.133)	0.633 (0.149)	0.758 (0.069)	0.723 (0.074)
	*t*-stat (*df* = 26)	2.88		2.37	
	*p*	0.008**		0.025*	
	Cohen’s *d*	0.55		0.46	
Flat prior	*M* (*SD*)	0.737 (0.142)	0.701 (0.157)	0.830 (0.052)	0.800 (0.067)
	*t*-stat (*df* = 26)	2.49		2.33	
	*p*	0.019*		0.028*	
	Cohen’s *d*	0.48		0.45	
Binomial prior	*M* (*SD*)	0.648 (0.13)	0.619 (0.14)	0.731 (0.061)	0.709 (0.074)
	*t*-stat (*df* = 26)	2.59		1.87	
	*p*	0.015*		0.073†	
	Cohen’s *d*	0.50		0.36	

** *p* < 0.01; *p < 0.05; ^†^*p* < 0.1.

**Table 4.** Results of within-subject comparisons of drug and placebo conditions for the atomoxetine group.

	Statistic	Expected utility		*p(correct)*	
		Placebo	Drug	Placebo	Drug
Learned prior	*M* (*SD*)	0.606 (0.118)	0.614 (0.166)	0.718 (0.051)	0.735 (0.076)
	*t*-stat (*df* = 21)	−0.23		−1.07	
	*p*	0.82		0.30	
	Cohen’s *d*	−0.05		−0.23	
Flat prior	*M* (*SD*)	0.690 (0.127)	0.683 (0.167)	0.815 (0.035)	0.817 (0.063)
	*t*-stat (*df* = 21)	0.40		−0.28	
	*p*	0.70		0.78	
	Cohen’s *d*	0.09		−0.06	
Binomial prior	*M* (*SD*)	0.597 (0.112)	0.6 (0.149)	0.708 (0.041)	0.721 (0.072)
	*t*-stat (*df* = 21)	−0.26		−1.2	
	*p*	0.80		0.25	
	Cohen’s *d*	−0.06		−0.26	

**Table 5.** Results of interaction of condition and drug type between groups.

	Statistic	Expected utility	*p(correct)*
Learned prior	*F*(1, 47)	4.73	5.98
	*p*	0.035*	0.018*
Flat prior	*F*(1, 47)	4.26	3.26
	*p*	0.045*	0.078^†^
Binomial prior	*F*(1, 47)	3.68	4.41
	*p*	0.061^†^	0.041*

* *p* < 0.05; ^†^*p* <0.1.

To compare findings with previous research and understand the causes of utility shifts demonstrated better, we also tested mean probabilities of correct choice at decision time between drug and placebo for each model (*p(correct)*; Clark et al., 2006). Pairwise *t*-tests showed significant differences between placebo and citalopram for learned and flat prior models – probabilities of correct decisions were lower under citalopram than placebo. The binomial prior model showed the same trend, although not reaching significance at the 0.05 level. It should be noted that the assumptions of the binomial prior model are not fully met in this variant of the IST, as the true underlying distribution of probabilities for each trial follow a truncated uniform distribution, as shown in the Methods. Once again, for atomoxetine, there were no significant differences, while the trend differences were in the opposite direction from citalopram.

Finally, we looked at the numbers of samples chosen and proportion of erroneous decisions, the latter defined as decisions choosing the minority colour or where the numbers of both colours were equal (as in this situation, taking an extra sample would increase expected utility). These are shown in Supplemental Table S4. There were no significant effects from either drug.

## Discussion

Acute serotonin reuptake inhibition reduced the expected utility of choices and probability of correct decisions in an information sampling context. Models showed similar effect sizes regardless of choice of model prior, and the effect on expected utility and probabilities were of similar magnitude. These effects were not observed following similar inhibition of the noradrenaline transporter, despite a similar historical side-effect profile of the treatments.

The modified task removes effects of temporal impulsivity and likely reduces effects of loss aversion. Risk preference was shown to be non-neutral from the RPE task in all but 5 out of 49 participants across the two groups, demonstrating the importance of the expected utility approach, which incorporates risk aversion, for modelling task behaviour. By computing individually parameterised expected utilities, our analysis allowed us to determine that the subjective value of decisions made under citalopram challenge was lower than those under placebo. This measure provides a stronger indication of changes to the effectiveness of sampling decisions than a probability measure alone, as it incorporates an independent measure of how individuals value certainty of reward. Additionally, by computing utility changes for three prior models representing feasible (but unknown) ways that participants may perceive the task contingencies, we demonstrate that this reduction occurs regardless of model choice and avoid the possibility of statistical inferences being biased by this unknown factor. We also computed and compared behaviour using the original *p(correct)* measure across the three prior models in order to facilitate comparisons with the existing literature and to demonstrate that the effect was reasonably robust without incorporating baseline risk aversion. Finally, the development of a new model with updating priors allows for the possibility that repeated trials may cause learning of task contingencies, and places minimal assumptions on this process.

Using a clinical dose of citalopram, we extend the findings of Crockett et al. (2012) and further our understanding of serotonin’s role in reflection impulsivity in the context of information sampling. The expected utility of choices and probability of being correct was lower on a clinical dose of citalopram, indicating a reduced tendency to be satisfied with an optimal amount of information to make a choice. This was consistent (maintaining significance or trend throughout) across the three tested models of participant priors, that is, whether participants maintained an unchanging assumption that the true distribution of numbers of colours was binomial or uniform, or if they learned and updated their prior with repeated trials. This finding does not appear to be driven by a simple strategy to take greater chances for a higher reward, which would have manifested in taking fewer samples. Instead, it indicates that citalopram alters another route to the use of sampled information. An example of this would be a lowered ability to adapt the number of samples taken to the Bayesian probability of correctness, given the information set. Alternatively, it could be due to an increased appetite for risk, based on information content. However, previous research with acute citalopram did not demonstrate changes in risk appetite on a gambling task (Macoveanu et al., 2013).

The effect of citalopram may be presynaptic or postsynaptic influences on serotonin at the synapse. Acute citalopram blockade of the serotonin transporter not only increases postsynaptic serotonin levels (David et al., 2003; Moret and Briley, 1996), but also increases serotonin availability at presynaptic autoreceptors that inhibit further release (Chaput et al., 1986; El Mansari et al., 2005; Nord et al., 2013). A clear consensus on the net effect has not been established. In our results, the effect of citalopram on decision probability was consistent with, and in the opposite direction to, that of ATD, as demonstrated by Crockett et al. By this, the effect may be postsynaptic enhancement of serotonin’s influence. However, ATD effects on learning tasks are not always the opposite effect of that observed by SRIs (Kanen et al., 2020).

There are alternative explanations, supporting a presynaptic account. Research by Chamberlain et al. (2006) and Skandali et al. (2018) showed probabilistic learning deficits from acute citalopram and escitalopram, where misleading feedback (i.e. feedback not in line with current contingencies, such as a loss when contingencies give rewards with 80% probability) was more likely to cause a shift of action than would be optimal. They posit that presynaptic serotonin autoreceptor activity may be responsible. Complementing this interpretation, Lottem et al. (2018) showed that activation of serotonergic neurons during foraging in mice promote exploitation of a rewarding patch rather than exploration of an alternative action. From a perspective of task switching and information gathering, our results are consistent with this presynaptic interpretation. Though the prepotent behaviour is to exploit in these studies and explore in the present, one speculative cohesive explanation may lie in covariation of serotonin activity and the amount of information required to instigate switch behaviour (from explore to exploit or vice versa). ATD studies of loss-chasing behaviour are consistent with this, where ATD increases switching out a sequence of gambles (and thereby accept the accumulated loss; Campbell-Meiklejohn et al., 2011). Serotonin may reduce the threshold required to switch or bias the calculation of choice utility (thereby suggesting a threshold has been reached earlier). To probe the presynaptic or postsynaptic mechanisms further and understand ATD effects on decision utility, a follow-up study using ATD with our methodology, as well as a study of longer-term treatment whereby post-synaptic effects dominate, would be beneficial.

While this study used single doses and was conducted on a healthy population, it nonetheless characterises the effects of the early stages of SRI treatment. Major depressive disorder itself presents with increased measures of reflection impulsivity (Tavares et al., 2007), and the issue becomes particularly pertinent where individuals show behaviours or co-morbidities with further effects on information sampling, such as the use of cannabis, amphetamines or opiates (Clark et al., 2006, 2009) and sufferers of narcolepsy with cataplexy (Delazer et al., 2011). If effects of SRI treatment on information sampling manifest similarly in these populations, this could lead to a possible additive effect and accentuate existing biases in the early stages of treatment. The longer-term clinical effects may depend on whether these effects of SRIs are sustained. Further research with chronic treatment and clinical populations would be needed to understand the clinical implications better.

Our null findings with atomoxetine are consistent with the Chamberlain et al. (2006) study. They showed that atomoxetine reduces motor impulsivity (failure to inhibit unwanted or premature motor actions), in line with noradrenergic and prefrontal dopamine influences on brain areas responsible for inhibitory signalling. Other research showed that atomoxetine reduces temporal impulsivity or delay discounting (choice of a smaller immediate reward over a larger delayed reward; Bizot et al., 2011). Both are distinct impulsivity subtypes dissociable from reflection impulsivity (Caswell et al., 2015). By contrast, directed exploratory decisions in the mIST task to seek out information for future gain are more deliberative, and the temporal aspect of reward delay was removed by design. While care must be taken in interpreting null results for atomoxetine, our results support a proposition that the distinction between these impulsivity types may be a pharmacological one.

### Limitations

A few limitations of the study design should be noted. The assignment to citalopram and atomoxetine studies was not randomised owing to data collection in independent experiments. Data were only blinded between placebo and drug conditions, not between the two experiments. This leads to the possibility that the two samples had characteristics that differed, aside from balanced age and sex. While effect sizes were convincing and benefited from within-subject designs, future studies and determination of clinical significance would benefit from a larger sample size.

The within-subjects design was chosen to orthogonalise any differences in the subject population from the effect of drug manipulation. A benefit is that drug comparisons are sensitive to effects that may be relevant to an individual, despite being small relative to variation across the population. Indeed, differences in placebo between the two groups were larger than the effect of citalopram. The atomoxetine group (compared to the citalopram group) showed, on trend, greater baseline risk seeking and lower baseline *p(correct)* and expected utility measures on the IST. This should be considered when interpreting effects in terms of variation of information sampling across a population.

The inclusion of the RPE measure was to account for risk aversion in our analysis of the IST. However, it was only measured at baseline. Therefore, drug effects on expected utility of IST choices could involve effects on tolerance to risk that were not captured in our study. Delineating effects on risk aversion from effects on the efficient use of sampled information would require a sensitive measure of risk aversion that could detect differences of risk aversion on and off the drug.

## Conclusion

We provide the first demonstration that a single dose of an SRI can impact information sampling, reflected in the utility of resulting decisions and probability of being correct. The findings of altered decision making with respect to optimal belief formation adds to our view of early-stage serotonergic treatment effects on exploration and choice. In a world where ever-growing quantities of information are available, an important choice is the choice to stop sampling and use the information at hand. Research on this decision continues to help us better understand the biology of choices in uncertain but information-rich environments. It is becoming ever clearer that serotonin plays a role in the shift from sampling the world to making a choice.

## Supplemental Material

sj-pdf-1-jop-10.1177_0269881121991571 – Supplemental material for Selective effects of serotonin on choices to gather more information Click here for additional data file. Supplemental material, sj-pdf-1-jop-10.1177_0269881121991571 for Selective effects of serotonin on choices to gather more information by James JA Livermore, Clare L Holmes, Jo Cutler, Maruša Levstek, Gyorgy Moga, James RC Brittain and Daniel Campbell-Meiklejohn in Journal of Psychopharmacology
