# Supplementary material for: Selective effects of serotonin on choices to gather more information
Source: J Psychopharmacol. 2021 Feb 18;35(6):631–40. doi: 10.1177/0269881121991571 (PMC8278551; doi:10.1177/0269881121991571)
Supplement: sj-pdf-1-jop-10.1177_0269881121991571 – Supplemental material for Selective effects of serotonin on choices to gather more information [file sj-pdf-1-jop-10.1177_0269881121991571.pdf]

Supplemental Methods

Bayesian inference on available information

Formalised as an inference problem, decision making requires a combination of displayed information (numbers of each colour) and, optimally, an inference on the likelihood of various proportions occurring. This is because the true range of proportions is not equiprobable across the potential proportions, but is constrained by an upper bound (proportions higher than a given level do not occur). The choice of prior results in quite different probabilities inferred from the available information, as shown in Axelsen et al. (2018) and Figure 4. This underscores the importance of being prior-agnostic in modelling.

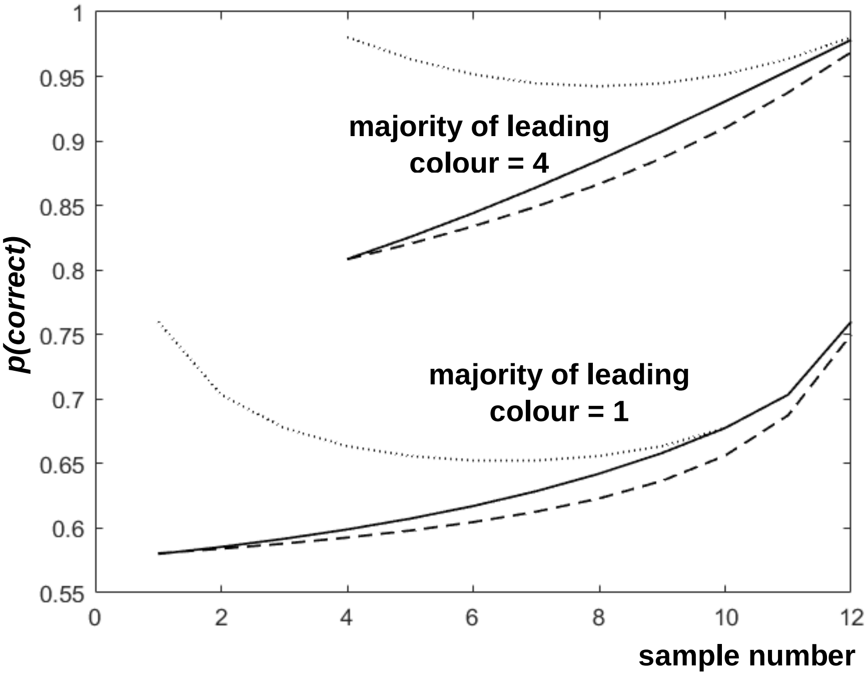

Figure S1. Probability of correct decision for each sample number, where solid lines show the underlying generative probabilities in our IST variant, dashed lines are probabilities inferred from a binomial prior, and dotted lines are inferred from a flat prior

Bayesian computations of decision probabilities

$$posterior = \frac{likelihood \times prior}{model\ evidence}$$

Bayes' theorem provides the method of using available information to compute the probability of an outcome. All three models use the hypergeometric distribution as likelihood (inference on current information), being the discrete probability distribution that describes the probability of a given number of outcomes when a fixed number of draws are made without replacement from a finite population. The models differ in their choice of priors or information that comes from before the current trial, such as previous experience from other trials.

Flat prior model (Bennett et al., 2016): this assumes that each combination of possible proportions of the majority to minority colour from 25:0 to 13:12 are equally likely, and that this assumption is maintained throughout the trials.

$$P(\theta|n_1, n_2) = \frac{\binom{\theta}{n_1}\binom{25-\theta}{n_2}}{\sum_{j=n_1}^{25-n_2}\binom{j}{n_1}\binom{25-j}{n_2}}$$

Binomial prior model (reformulated by Axelsen et al. (2018), equal to the original *P(correct)* measure in Clark et al., 2006): this assumes a personal prior on the underlying generative process of p=0.5, i.e. that on average each colour is equally likely, resulting in a binomial distribution on the two colours. Notably, this means that extreme values of proportions are considered much less likely than values where the majority and minority colours have similar numbers.

$$P(\theta|n_1, n_2) = \frac{\binom{\theta}{n_1}\binom{25-\theta}{n_2}\binom{25}{\theta}}{\sum_{j=n_1}^{25-n_2}\binom{j}{n_1}\binom{25-j}{n_2}\binom{25}{j}}$$

Learned prior model, developed in this paper: this assumes that on trial T, information about true proportions given in the feedback for trials 1 to T-1 are incorporated in the form of a categorical distribution, where observed numbers of proportions are assigned a probability according to the number of times they were observed, and unobserved proportions are assigned a zero probability. Only trials $T \geq 2$ are considered for analysis.

$$C_M = \sum_{t=1}^{T-1} I_t \ \ where\ I_t \begin{cases} 1 \text{ if } \theta = M \\ 0 \text{ otherwise} \end{cases}$$

$$P(\theta|n_1, n_2) = \frac{\binom{\theta}{n_1}\binom{25-\theta}{n_2}\frac{C_M}{2(T-1)}}{\sum_{j=n_1}^{25-n_2}\binom{j}{n_1}\binom{25-j}{n_2}\sum_{\Theta=0}^{25}P(\Theta|n_1, n_2)} \text{ where } T \geq 2$$

All models then compute the probability of a correct decision by summing probabilities that the proportion of the chosen colour is 13 or higher, i.e. that the chosen colour is in the majority.

$$P(correct) = P(\theta \geq 13|n_1, n_2) = \sum_{M=13}^{25} P(\theta = M|n_1, n_2)$$

Mediation analysis

Where significant drug effects on measures were found on the mIST but differences in VAS scores existed between drug and placebo conditions, we conducted a supplementary analysis to determine whether self-reported drug effects may mediate some of the drug effects. This was conducted using the MEMORE macro in SPSS (MEMORE v2.1; Montoya 2018). Figure S2 shows the paths of possible mediation.

Figure S2. Paths of mediation

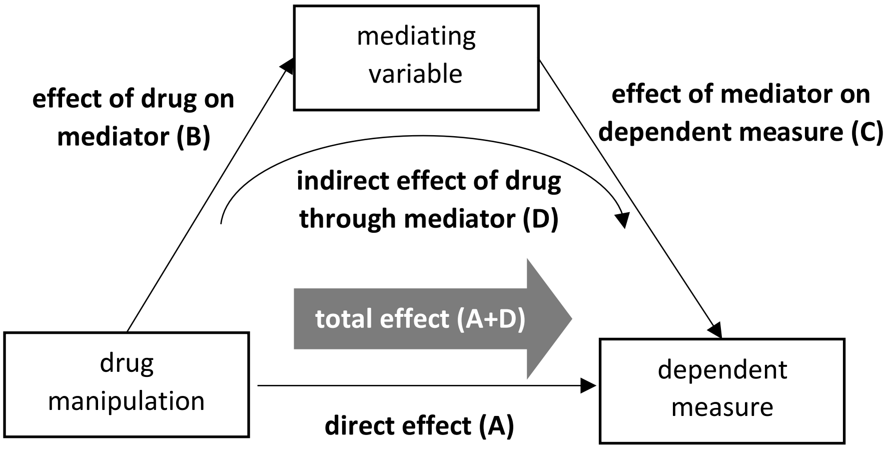

Supplemental Results

Coefficient of variation analysis

Table S1. Results of coefficient of variation analysis

measure	CITALOPRAM GROUP mean (SD)	ATOMOXETINE GROUP mean (SD)
p(correct)	0.168 (0.053)	0.164 (0.045)
expected utility	0.197 (0.083)	0.203 (0.074)
sample number	0.275 (0.090)	0.353 (0.169)

Table S1 shows results of the coefficient of variation analysis. Coefficients of variation were lower for both *p(correct)* and expected utility than sample number in both the citalopram group (*p(correct):* $t(26) = 5.08$, $p < .001$; expected utility: $t(26) = 3.38$, $p = .002$) and the atomoxetine group (*p(correct):* $t(22) = 5.27$, $p < .001$; expected utility: $t(22) = 3.81$, $p = .001$). Thus, both measures were more consistent between trials than the sample number measure.

Comparison with literature using the original IST

Table S2. Results of previous studies using the original IST task

Reference	Group size	Sample number mean (SD)
(Clark et al., 2006)	26	7.5 (2.8)
(Tavares et al., 2007)	25	9.5 (3.7)
(Chamberlain et al., 2007)	20	7.5 (3.0)
(Clark, Roiser, Robbins, & Sahakian, 2009)	19	8.9 (2.5)
(Delazer et al., 2011)	58	9.6 (4.3)

We searched the literature for data using the IST on healthy participants, excluding those using either an adolescent or older-aged subject population, or where the standard deviation of sampling decisions was unavailable. Where patient and control groups were tested, only control group statistics were used. Five studies were located including the original paper that introduced the task, which are shown in Table S2. The weighted mean sample number was 8.84.

Testing VAS differences

Table S3. VAS score comparisons at test time, with scores on a 100-point scale. For antonym pairs, higher numbers are closer to the second term. P - placebo condition, D – drug condition, * p < .05

	CITALOPRAM GROUP				ATOMOXETINE GROUP				BETWEEN GROUPS	
**scale**	**placebo mean (SD)**	**drug mean (SD)**	***t*-stat (df=26)**	***p***	**placebo mean (SD)**	**drug mean (SD)**	***t*-stat (df=21)**	***p***	***t*-stat (df=47)**	***p***
**nausea**	4.32 (6.63)	8.24 (11.5)	-2.06	.050	3.36 (3.94)	9.91 (14.0)	-2.31	**.031***	-0.79	.43
**headache**	10.8 (15.9)	11.26 (16.8)	-0.18	.86	4.39 (4.40)	7.66 (12.6)	-1.49	.15	-0.85	.40
**dizziness**	7.28 (12.7)	8.89 (8.7)	-1.01	.32	5.16 (5.66)	9.55 (11.8)	-2.00	.058	-1.05	.30
**alert – drowsy**	36.8 (17.0)	41.2 (18.8)	-1.15	.26	43.9 (14.3)	45.6 (18.5)	-0.377	.71	0.44	.66
**stimulated – sedated**	41.7 (13.3)	44.2 (13.7)	-0.91	.37	46.4 (11.4)	47.7 (14.4)	-0.367	.72	0.27	.79
**restless – peaceful**	65.3 (13.9)	62.7 (18.7)	0.80	.43	63.8 (12.0)	60.4 (17.3)	1.25	.23	0.19	.85
**irritable – good-humoured**	64.7 (15.4)	66.6 (14.5)	-0.76	.45	70.9 (13.9)	65.6 (13.7)	1.84	.08	1.88	.066
**anxious – calm**	70.8 (13.3)	68.7 (14.6)	0.63	.53	70.6 (13.8)	68.1 (15.0)	0.95	.35	0.10	.92

Table S4. Results of sample number and erroneous decisions

	CITALOPRAM GROUP				ATOMOXETINE GROUP			
	**placebo mean (SD)**	**drug mean (SD)**	***t*-stat (df=26)**	***p***	**placebo**	**drug**	***t*-stat (df=21)**	***P***
**sample no**	8.71 (3.52)	8.73 (3.45)	0.036	.97	7.91 (2.98)	8.42 (3.82)	1.03	.32
**erroneous decisions**	0.062 (0.077)	0.074 (0.093)	0.65	.52	0.053 (0.067)	0.074 (0.081)	1.07	.29

Mediation analysis

As nausea score differences were at the threshold of significance in the citalopram group, we carried out a mediation analysis incorporating the change of nausea scores at test time, in each condition, as a mediating variable.

Table S4. Results of mediation analysis for VAS effects of nausea on mIST outcome variables. † in units of the dependent measures, ‡ in units of the VAS score. Paths refer to the mediation paths shown in Figure S2. Bolded figures are where the confidence intervals does not overlap zero (indicating a significant effect)

	expected utility			*p(correct)*		
**effect**	**learned prior mean effect [95% CI]**	**flat prior mean effect [95% CI]**	**binomial prior mean effect [95% CI]**	**learned prior mean effect [95% CI]**	**flat prior mean effect [95% CI]**	**binomial prior mean effect [95% CI]**
**total citalopram effect on dependent measure (paths A+D) †**	**-0.038 [-0.066, -0.011]**	**-0.036 [-0.066, -0.006]**	**-0.029 [-0.052, -0.006]**	**-0.035 [-0.065, -0.006]**	**-0.029 [-0.056, -0.004]**	**-0.022 [-0.045, -0.002]**
**direct citalopram effect on dependent measure (path A) †**	**-0.045 [-0.076, -0.014]**	**-0.044 [-0.078, -0.011]**	**-0.037 [-0.063, -0.011]**	-0.030 [-0.063, 0.004]	-0.024 [-0.054, 0.006]	-0.015 [-0.041, 0.012]
**effect of citalopram on nausea (path B) ‡**	3.93 [-0.002, 7.85]	3.93 [-0.002, 7.85]	3.93 [-0.002, 7.85]	3.93 [-0.002, 7.85]	3.93 [-0.002, 7.85]	3.93 [-0.002, 7.85]
**effect of nausea on dependent measure (path C) †**	0.002 [-0.002, 0.005]	0.002 [-0.002, 0.006]	0.002 [-0.008, 0.005]	-0.001 [-0.005, 0.002]	-0.002 [-0.005, 0.002]	-0.002 [-0.005, 0.001]
**indirect effect of citalopram on dependent measure through nausea (path D) †**	0.007 [-0.018, 0.029]	0.008 [-0.020, 0.029]	0.008 [-0.010, 0.025]	-0.006 [-0.020, 0.030]	-0.006 [-0.023, 0.030]	-0.007 [-0.016, 0.023]

Table S4 shows the results of this analysis. Nausea did not predict any dependent measure (path C: all confidence intervals overlap zero), meaning that it was very unlikely that nausea mediated effects of drugs on mIST behaviour. All indirect effects of citalopram through nausea were small and confidence intervals overlapped zero, confirming no mediation. Given these null effects and given that nausea was not hypothesised to alter mIST behaviour, total effects were used for inference and subsequent analyses. However, it may be noted that when changes of nausea are included as covariates in the analysis of citalopram effects, expected utility measures show stronger

effects than without. In contrast, when nausea changes are included in the *p(correct)* analysis, effects trend in the same direction as the main analysis, but direct effects have 95% confidence intervals that overlap with zero (likely due to increased demands for statistical power with this variable included). No changes of statistical inference could be made from mediation analysis of ATX data (all direct and indirect effects were non-significant).

Supplemental References

Axelsen MC, Jepsen JRM and Bak N (2018) The Choice of Prior in Bayesian Modeling of the Information Sampling Task. *Biological Psychiatry* 83(12). Elsevier: e59–e60. DOI: 10.1016/J.BIOPSYCH.2017.04.021.

Bennett D, Oldham S, Dawson A, et al. (2016) Systematic Overestimation of Reflection Impulsivity in the Information Sampling Task. *Biological Psychiatry*. DOI: 10.1016/j.biopsych.2016.05.027.

Chamberlain SR, Hampshire A, Müller U, et al. (2009) Atomoxetine Modulates Right Inferior Frontal Activation During Inhibitory Control: A Pharmacological Functional Magnetic Resonance Imaging Study. *Biological Psychiatry* 65(7). Attention Deficit/Hyperactivity Disorder: From Circuit Dysfunction to Novel Treatments: 550–555. DOI: 10.1016/j.biopsych.2008.10.014.

Clark L, Robbins TW, Ersche KD, et al. (2006) Reflection Impulsivity in Current and Former Substance Users. *Biological Psychiatry* 60(5): 515–522. DOI: 10.1016/j.biopsych.2005.11.007.

Clark L, Roiser J, Robbins T, et al. (2009) Disrupted `reflection' impulsivity in cannabis users but not current or former ecstasy users. *Journal of Psychopharmacology* 23(1). SAGE PublicationsSage UK: London, England: 14–22. DOI: 10.1177/0269881108089587.

Delazer M, Högl B, Zamarian L, et al. (2011) Executive functions, information sampling, and decision making in narcolepsy with cataplexy. *Neuropsychology* 25(4): 477–487. DOI: 10.1037/a0022357.

Montoya, A.K. (2019) Moderation analysis in two-instance repeated measures designs: Probing methods and multiple moderator models. *Behavior Research Methods*, 51(1): 61-82. DOI: 10.3758/s13428-018-1088-6.

Tavares JVT, Clark L, Cannon DM, et al. (2007) Distinct Profiles of Neurocognitive Function in Unmedicated Unipolar Depression and Bipolar II Depression. *Biological Psychiatry* 62(8). Elsevier: 917–924. DOI: 10.1016/J.BIOPSYCH.2007.05.034.
